# Supplementary material for: Reduction of Ethyl Carbamate in an Alcoholic Beverage by CRISPR/Cas9-Based Genome Editing of the Wild Yeast
Source: Foods. 2022 Dec 25;12(1):102. doi: 10.3390/foods12010102 (PMC9818936; doi:10.3390/foods12010102)
Supplement: Supplementary file 1 [file foods-12-00102-s001.zip › foods-2104687-supplementary.pdf]

Supplementary Figures

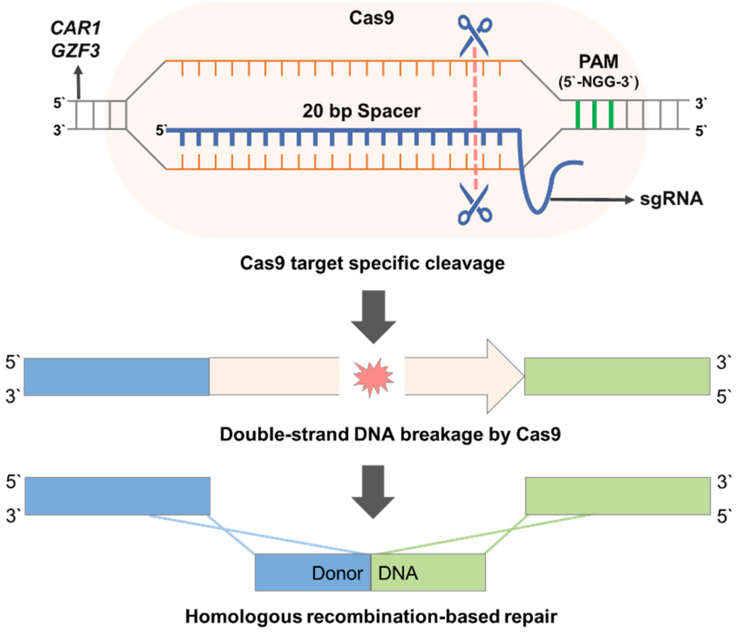

Figure S1. Diagram of the *CAR1* and *GZF3* gene deletion using CRISPR/Cas9 system. Through combination with sgRNA, the Cas9 recognizes 20 bp containing PAM sequence and causes target specific cleavage. After double-strand DNA breakage, homologous recombination-based repair is performed by Donor DNA.

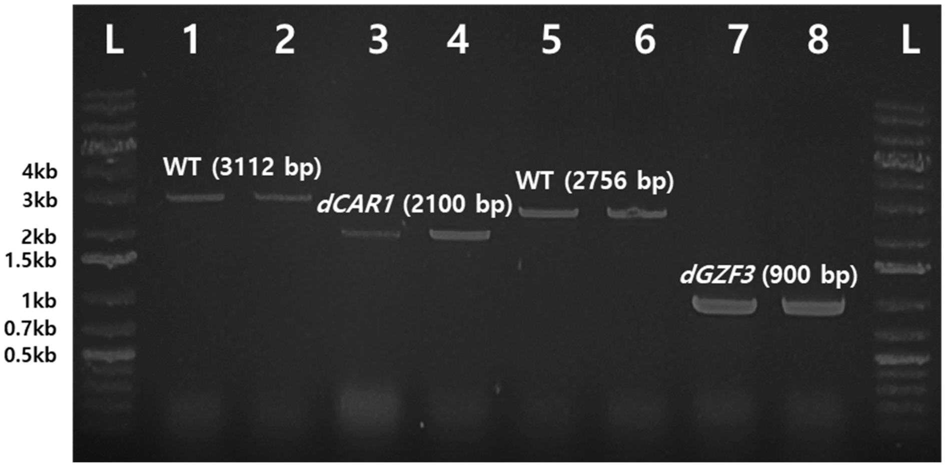

Figure S2. Agarose gel electrophoresis (1% agarose) of colony PCR products. Lanes 1 and 2 are Wild-Type yeast colony band for check *CAR1* gene deletion. When *CAR1* gene is deleted, a 2.1 kb band appears. Lanes 5 and 6 are Wild-Type yeast colony band for check *GZF3* gene deletion. When *GZF3* gene is deleted, a 0.9 kb band appears. Lane L is DNA ladder.

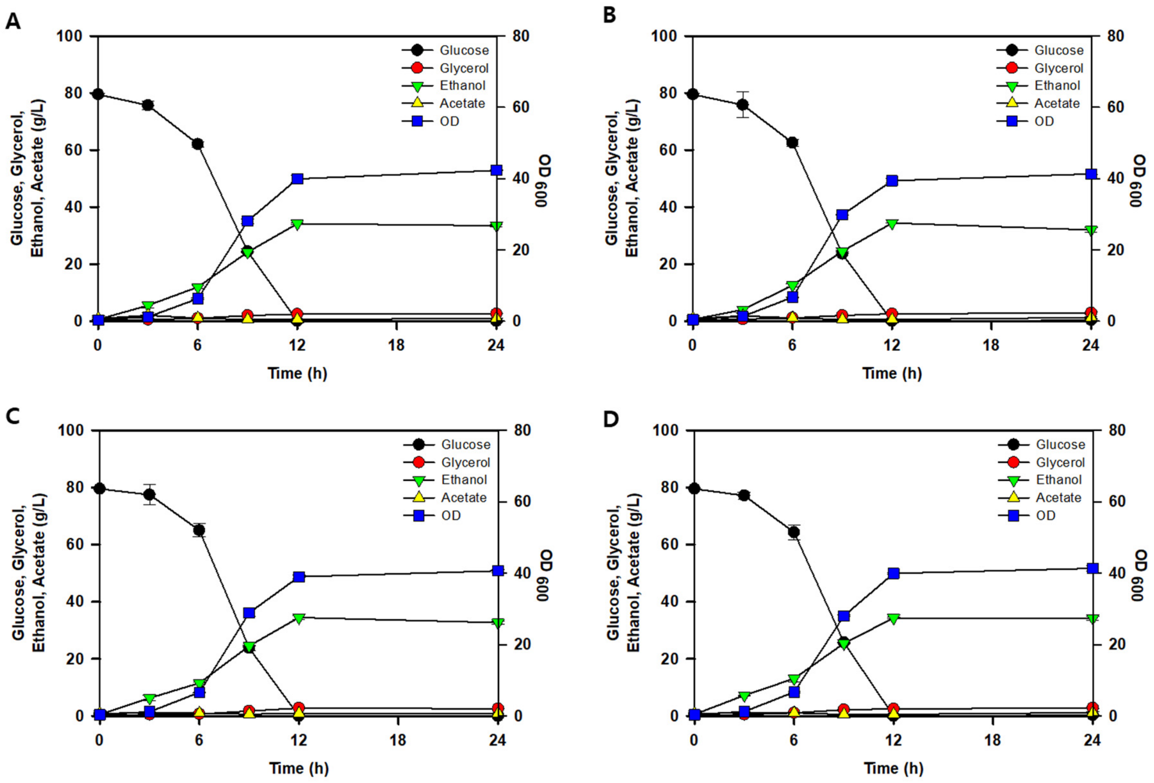

Figure S3. **Batch fermentation profiles of *S. cerevisiae* GRL6, *dCAR1*, *dGZF3*, and *dCAR1&GZF3* in the YPD8 medium containing 80 g/L glucose with 50 mg/L urea and 50 mM arginine for 24 h.** (A) Fermentation profile of *S. cerevisiae* GRL6, (B) Fermentation profile of *S. cerevisiae dCAR1*, (C) Fermentation profile of *S. cerevisiae dGZF3*, (D) Fermentation profile of *S. cerevisiae dCAR1&GZF3*.

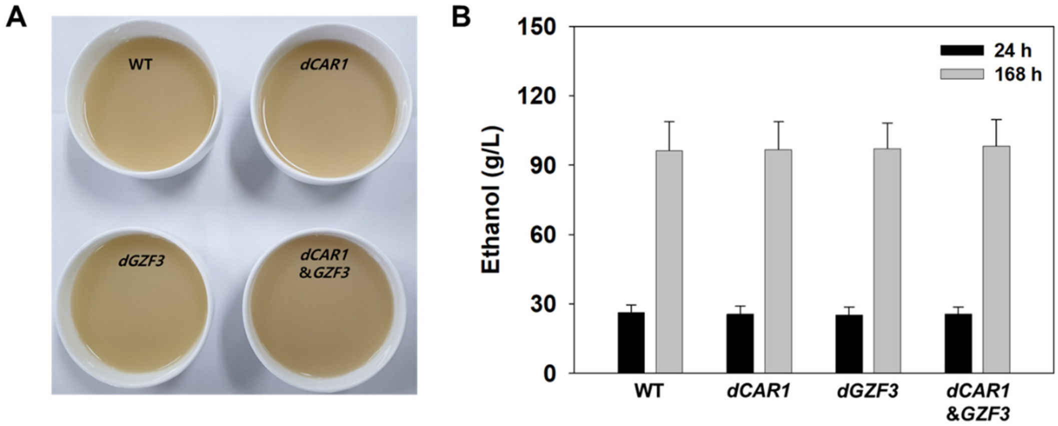

Figure S4. Brewing alcoholic beverages by using the engineered *S. cerevisiae* as a starter culture. (A) *Makgeolli* that brewed with the *S. cerevisiae* GRL6, *dCAR1*, *dGZF3*, and *dCAR1&GZF3*. (B) Levels of ethanol in *Makgeolli* brewed with the *S. cerevisiae* GRL6, *dCAR1*, *dGZF3*, and *dCAR1&GZF3*.

Supplementary Tables

Table S1. Comparison of the fermentation capability in batch fermentation at 12 h by the wild-type *S. cerevisiae GRL6* and the engineered *S. cerevisiae dCAR1*, *dGZF3*, and *dCAR1&GZF3*

Strains	Final OD_{600}	Ethanol (g/L)	Ethanol yield (g ethanol/g glucose)	Ethanol productivity (g/L/h)
S. cerevisiae GRL6	39.9 ± 1.0	34.2 ± 0.2	0.43 ± 0.00	2.85 ± 0.02
Δ*CAR1*	41.3 ± 0.7	34.4 ± 0.1	0.44 ± 0.01	2.87 ± 0.01
Δ*GZF3*	40.7 ± 0.6	34.5 ± 0.6	0.44 ± 0.01	2.87 ± 0.05
Δ*CAR1&GZF3*	41.4 ± 0.3	34.3 ± 0.1	0.44 ± 0.01	2.86 ± 0.01

Table S2. Comparison of the ethyl carbamate concentration in batch fermentation by the wild-type *S. cerevisiae GRL6* and the engineered *S. cerevisiae dCAR1*, *dGZF3*, and *dCAR1&GZF3*

Strains	Ethyl carbamate concentration (μg/L)			Reduction rate compared to *S. cerevisiae* GRL6 (%)		
	0 h	24 h	168 h	0 h	24 h	168 h
S. cerevisiae GRL6	13.2 ± 1.2	41.2 ± 5.2	56.9 ± 1.8	0	0	0
Δ*CAR1*	13.1 ± 1.5	28.0 ± 2.7	42.1 ± 0.9	0.7	32.1	26.1
Δ*GZF3*	13.0 ± 0.8	34.2 ± 2.9	48.2 ± 3.8	1.7	16.9	15.3
Δ*CAR1&GZF3*	12.9 ± 1.2	19.7 ± 5.1	37.6 ± 2.1	2.0	52.1	33.9

Table S3. Comparison of the ethyl carbamate concentration in *Makgeolli* fermentation by the wild-type *S. cerevisiae GRL6* and the engineered *S. cerevisiae dCAR1*, *dGZF3*, and *dCAR1&GZF3*

Strains	Ethyl carbamate concentration (μg/L)			Reduction rate compared to *S. cerevisiae* GRL6 (%)		
	0 h	24 h	168 h	0 h	24 h	168 h
S. cerevisiae GRL6	17.4 ± 2.0	30.2 ± 5.0	42.3 ± 6.5	0	0	0
Δ*CAR1*	17.8 ± 1.3	22.2 ± 3.3	35.2 ± 8.0	-2.1	26.5	16.6
Δ*GZF3*	20.5 ± 5.1	26.7 ± 5.9	36.3 ± 7.0	-17.6	11.5	14.0
Δ*CAR1&GZF3*	20.6 ± 1.4	19.0 ± 2.2	24.7 ± 4.5	-18.5	37.0	41.6
